# Serum and molecular assessment of vascular endothelial growth factor (VEGF) and hypoxia-inducible factor 1α (HIF-1α) in canine mammary tumors

**DOI:** 10.1186/1753-6561-7-S2-P49

**Published:** 2013-04-04

**Authors:** Marina G Moschetta, Gabriela B Gelaleti, Larissa B Maschio, Bruna V Jardim, Camila Leonel, Lívia C Ferreira, Juliana R Lopes, Naiane N Gonçalves, Gustavo R Martins, Thaíz F Borin, Debora APC Zuccari

**Affiliations:** 1 Departament of Molecular Biology, Faculdade de Medicina de São José do Rio Preto - FAMERP, São José do Rio Preto, SP, Brazil; 2 Department of Biology, Universidade Estadual Paulista - UNESP/IBILCE, São José do Rio Preto, SP, Brazil; 3 Departament of Molecular Biology – Laboratory of Molecular Cancer Investigation, Faculdade de Medicina de São José do Rio Preto - FAMERP, São José do Rio Preto, SP, Brazil

## Background

Due to the many similarities shared by humans and dogs, canine mammary tumors are an excellent model to comprehend various aspects of mammary neoplasias. The tumor cells have the ability to promote changes in their functionality in order to survive. In situations of hypoxia, tumor cells produce and release pro and antiangiogenic factors that regulate the process of angiogenesis. The hypoxia-inducible factor 1 (HIF -1) is a central regulator of pathophysiological response of mammalian cells to low oxygen levels, able to activate transcription of the gene that promotes the induction of vascular endothelial growth factor (VEGF),which in turn, promotes angiogenesis through its ability to stimulate growth, migration and invasion of endothelial cells, leading to the formation of new blood vessels and subsequent tumor growth. In this context, the aim of this study was to measure serum levels of VEGF and HIF-1 and to relate with clinicopathological parameters and survival.

## Patients and methods

Through enzyme-linked immunosorbent assay and qPCR were evaluated and statistically related 30 female dogs with mammary tumor and 47 controls.

## Results

High levels of VEGF were correlated with abundant irrigation (p=0.02), metastase (p=0.003), death (p=0.001) and low survival (p<0.0001); however HIF-1 levels was not related with clinicopathological features investigated. The VEGF was superexpressed in tumors with abundant irrigation and also in female dogs with metastase, recidive and death while the HIF-1α was underexpressed.

## Conclusions

Our results show that these proteins play an important role in angiogenesis and are useful in predicting tumor progression in canine mammary tumors.

## Financial support

FAPESP.

